# An epidemiological model for proliferative kidney disease in salmonid populations

**DOI:** 10.1186/s13071-016-1759-z

**Published:** 2016-09-05

**Authors:** Luca Carraro, Lorenzo Mari, Hanna Hartikainen, Nicole Strepparava, Thomas Wahli, Jukka Jokela, Marino Gatto, Andrea Rinaldo, Enrico Bertuzzo

**Affiliations:** 1 Laboratory of Ecohydrology, École Polytechnique Fédérale de Lausanne, Station 2, Lausanne, 1015 Switzerland; 2 Dipartimento di Elettronica, Informazione e Bioingegneria, Politecnico di Milano, Via Ponzio 34/5, Milan, 20133 Italy; 3 Eawag, Swiss Federal Institute of Aquatic Science and Technology, Überlandstrasse 133, Dübendorf, 8600 Switzerland; 4 Institute of Integrative Biology, ETH Zürich, Universitätstrasse 16, Zürich, 8092 Switzerland; 5 Centre for Fish and Wildlife Health, Universität Bern, Länggassstrasse 122, Bern, 3012 Switzerland; 6 Dipartimento di Ingegneria Civile, Edile ed Ambientale, Università di Padova, Via Marzolo 9, Padova, 35131 Italy

**Keywords:** Discrete-continuous hybrid model, Climate change, Disease ecology, *Fredericella sultana*

## Abstract

**Background:**

Proliferative kidney disease (PKD) affects salmonid populations in European and North-American rivers. It is caused by the endoparasitic myxozoan *Tetracapsuloides bryosalmonae*, which exploits freshwater bryozoans and salmonids as hosts. Incidence and severity of PKD in brown trout populations have recently increased rapidly, causing a decline in fish catches and local extinctions in many river systems. PKD incidence and fish mortality are known to be enhanced by warmer water temperatures. Therefore, environmental change is feared to increase the severity of PKD outbreaks and extend the disease range to higher latitude and altitude regions. We present the first mathematical model regarding the epidemiology of PKD, including the complex life-cycle of its causative agent across multiple hosts.

**Methods:**

A dynamical model of PKD epidemiology in riverine host populations is developed. The model accounts for local demographic and epidemiological dynamics of bryozoans and fish, explicitly incorporates the role of temperature, and couples intra-seasonal and inter-seasonal dynamics. The former are described in a continuous-time domain, the latter in a discrete-time domain. Stability and sensitivity analyses are performed to investigate the key processes controlling parasite invasion and persistence.

**Results:**

Stability analysis shows that, for realistic parameter ranges, a disease-free system is highly invasible, which implies that the introduction of the parasite in a susceptible community is very likely to trigger a disease outbreak. Sensitivity analysis shows that, when the disease is endemic, the impact of PKD outbreaks is mostly controlled by the rates of disease development in the fish population.

**Conclusions:**

The developed mathematical model helps further our understanding of the modes of transmission of PKD in wild salmonid populations, and provides the basis for the design of interventions or mitigation strategies. It can also be used to project changes in disease severity and prevalence because of temperature regime shifts, and to guide field and laboratory experiments.

**Electronic supplementary material:**

The online version of this article (doi:10.1186/s13071-016-1759-z) contains supplementary material, which is available to authorized users.

## Background

Epidemics of emerging diseases may have large economic and ecological impacts, threaten livelihoods, elicit biodiversity losses and affect key ecosystem services. Understanding causes and patterns of disease emergence becomes even more topical as causative and correlative links with global climate and environmental change are established [1–3]. Proliferative Kidney Disease (PKD) of salmonid fish, caused by *Tetracapsuloides bryosalmonae* (phylum Cnidaria, class Malacosporea) [4], is highly problematic for hatcheries and fish farms, potentially reaching 100 % infection prevalence and high mortalities (up to 95 %) in affected fish [5, 6]. Although the impacts of PKD in wild fish populations are still poorly known, PKD is considered a key factor contributing to the decline of wild salmonid populations in Switzerland and Northern Europe [7–9].

The parasite life-cycle alternates between freshwater bryozoans, as primary hosts [10–12], and salmonid fish, where infection can cause PKD [4, 13]. The transmission between hosts occurs through spores that are released into water. Development and pathology of PKD are dependent on water temperature, as clinical signs and disease-related mortality increase with increasing water temperatures [4, 5, 9, 14–16]. Water temperature is also key to the development of *T. bryosalmonae* in its bryozoan host. Increased temperatures have been shown to promote the production of spores infective to fish [17, 18]. The connections between temperature, disease development and fish mortality suggest that, due to climate change, the prevalence, severity and distribution of PKD are likely to increase further [19]. Hence, PKD must be considered as an emerging disease having a sizeable and growing impact on the health of salmonid populations and, consequently, on the economy of fish farming industry and hatcheries. In the perspective of understanding drivers and controls of the disease, and in the search for mitigation strategies, the development of a dynamical model of PKD transmission becomes crucial.

Epidemiological models incorporating parasites with simple life-cycles have a long history [20, 21] and have been successfully applied to several human diseases and zoonoses. Conversely, models incorporating parasites and pathogens with complex life-cycles and multiple hosts are less frequently developed, although many major human and wildlife diseases are caused by parasites with such life-cycles. Successful application of epidemiological models for these diseases fundamentally relies on the incorporation of the ecological dynamics of the different host populations. In the context of fish diseases, epidemiological models have been exploited to address various issues, such as sea lice infection on salmon farms [22]; Koi herpes virus of the common carp (*Cyprinus carpio*) [23]; amyloodinosis, a disease of warm water mariculture [24]; fish-borne zoonotic trematodes in agriculture-aquaculture farms [25]; *Ceratomyxa shasta*, a myxozoan parasite endemic to river systems throughout the Pacific northwest region of North America [26]; and whirling disease, a myxozoan disease of farmed and wild salmonids [27]. As for PKD, the only modeling attempt used a Bayesian probability network to assess the decline of brown trout, citing PKD as a driver of increased mortality [28].

The present work proposes an epidemiological model capable of describing the intra- and inter-annual dynamics of PKD. The model aims to translate the current knowledge of the modes of transmission of the disease and of the life-cycle of its causative agent into a mathematical form, making the connection between temperature and epidemiological parameters explicit. In this way, it is possible to state under which conditions PKD can establish in a fish population. The development and the analysis of such a model is also instrumental to guiding further experimental and field studies on PKD.

## Methods

In this section, we first describe the transmission cycle of PKD, and subsequently present the mathematical model that stems from such biological and epidemiological evidence.

### PKD transmission cycle

*Tetracapsuloides bryosalmonae* has a complex life-cycle that exploits freshwater bryozoans and salmonids as hosts (for review, see [29]). Within bryozoans, the parasite can express either covert or overt infection stages. During covert infections, the parasite exists as non-virulent single-cell stages [30, 31]. The transition to overt infection implies increase in virulence, with the formation of multicellular sacs from which thousands of *T. bryosalmonae* spores (approximately 20 μm in diameter) are released into water. Spores are characterized by two amoeboid cells and four polar capsules [32, 33]. Parasite transmission from bryozoans to fish can thus take place only during the overt infection stage. Overt infection hampers bryozoan growth, whereas covert stages pose low energetic cost to bryozoans [17]. Peaks of overt infection have been observed in late spring and autumn [34]. In particular, overt stages develop when bryozoans are undergoing enhanced growth as a result of optimal temperatures or food levels [18, 35]. Overt infection also elicits temporary castration [36], with a severe reduction in the production of statoblasts (asexually produced dormant propagules). Covertly infected bryozoans can produce infected statoblasts thus allowing vertical transmission of *T. bryosalmonae* [37]. Recovery mechanisms for bryozoan colonies are poorly observed and understood [37].

Parasite spores released into water by overtly infected bryozoans infect fish through skin and gills [38, 39]. *Tetracapsuloides bryosalmonae* subsequently enters the kidney of fish hosts, where it undergoes multiplication and differentiation from extrasporogonic to sporogonic stages [40]. Spores developed in the lumen of kidney tubules contain one amoebid cell and two polar capsules [41], and are eventually excreted via urine [42]. Although PKD seems to develop in all salmonids to a varying degree, life-cycle completion may be highly species-specific. For example, in Europe, parasite spores infective to bryozoans develop in the brown trout (*Salmo trutta*) and the brook trout (*Salvelinus fontinalis*), but not in the rainbow trout (*Oncorhynchus mykiss*) [43–45]. Fish infected with PKD often die owing to secondary infections [13]; however, PKD alone has been shown to cause mortality [14, 15]. Fish that do not die during the acute phase of the infection may become long-term carriers of the parasite. Such carriers are reportedly able to infect *Fredericella sultana*, one of the most common bryozoan hosts of *T. bryosalmonae*, for a period up to 2 years after exposure [43].

### Model

**Fig. 1:**
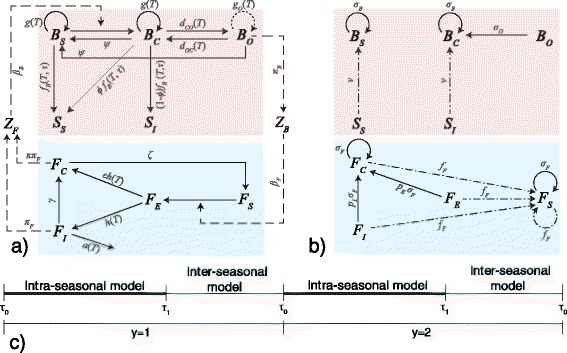
Schematic representation of PKD transmission dynamics. **a** Within-season dynamics. The red-shaded area highlights the bryozoan sub-model. The circular arrows illustrate bryozoan growth. The dotted line indicates that the growth of overtly infected bryozoans is impaired by the parasite. The blue-shaded area highlights the fish sub-model. The dead-end arrow refers to excess PKD-induced fish mortality. In both sub-models, straight solid lines indicate fluxes among epidemiological classes, dashed arrows represent disease transmission between the two sub-systems. Natural mortality is not displayed. Parameters driving the transition between classes are displayed in gray. Letters *T* and *τ* indicate fluxes that are expected to be dependent on water temperature *T* and time *τ*. **b** Schematic representation of between-season (overwintering) dynamics relevant to PKD transmission. Colors as in (**a**). Continuous lines represent survival over winter. Dashed-dotted lines stand for reproduction processes. **c** Graphic timeline of the discrete-continuous hybrid model

The proposed model couples PKD transmission and population dynamics of fish and bryozoan populations. When reproduction processes are concentrated in time (as in our case, in which spawning and hatching for fish, and statoblast hatch for bryozoans occur mainly during the cold season), population dynamics are traditionally analyzed with discrete-time models (see the widely known Ricker model [46], introduced to study stock and recruitment in fisheries). On the other hand, PKD transmission occurs continuously throughout the warm season, thus calling for a continuous modelling approach (e.g. the classical Susceptible-Infected-Recovered model [20]). We therefore propose a discrete-continuous hybrid framework that couples intra-seasonal and inter-seasonal dynamics. Seasons can be thought of as the periods of bryozoan proliferation (e.g. from April to November, depending on climate). Within-season dynamics are described by a set of coupled ordinary differential equations expressing how bryozoan biomass, fish density, and the abundances of infective spores and statoblasts change during the warm season. The transition between the end of a warm season and the beginning of the next one is modelled as a discrete-time update. Therefore, between-season dynamics are described by a set of difference equations. An interesting framework to formalize discrete-continuous hybrid models is the so-called time scale calculus [47]; however, the complexity of the model presented in the following paragraphs prevents an effective use of such formalism. In this work, we focus on a local-scale model, able to mimic PKD dynamics in an isolated water body, where fish and bryozoans are well mixed, and there are no additional inputs or outputs. An outline of the model is shown in Fig. 1. The variables of the model are listed in Table 1; all state variables are referred to as concentrations.

**Table 1:** List of model variables

Symbol	Variable^a^	Dimension
*B*_*S*_	Biomass of susceptible bryozoans	[ML^-3^]
*B*_*C*_	Biomass of covertly infected bryozoans	[ML^-3^]
*B*_*O*_	Biomass of overtly infected bryozoans	[ML^-3^]
*S*_*S*_	Non-infected statoblast abundance	[L^-3^]
*S*_*I*_	Infected statoblast abundance	[L^-3^]
*F*_*S*_	Susceptible fish abundance	[L^-3^]
*F*_*E*_	Exposed fish abundance	[L^-3^]
*F*_*I*_	Infected fish abundance	[L^-3^]
*F*_*C*_	Carrier fish abundance	[L^-3^]
*Z*_*B*_	Abundance of spores released by bryozoans	[L^-3^]
*Z*_*F*_	Abundance of spores released by fish	[L^-3^]
${Z}_B^{*}={\beta}_F{Z}_B$	Abundance of equivalent spores released by bryozoans	[T^-1^]
${Z}_F^{*}={\beta}_B{Z}_F$	Abundance of equivalent spores released by fish	[T^-1^]

^a^All model variables are referred to a water volume

#### Within-season model

The dynamics of the state variables during the warm season (from time *τ*_0_ to *τ*_1_) of year *y* (see Fig. 1c) is described by the following system of first-order differential equations:

1a $$ \frac{d{B}_S}{d\tau }={g}_S\left({B}_S,{B}_C,{B}_O;T\right){B}_S-{\beta}_B{Z}_F{B}_S+\psi\;\left({B}_C+{B}_O\right); $$

1b $$ \frac{d{B}_C}{d\tau }={g}_C\left({B}_S,{B}_C,{B}_O;T\right){B}_C+{\beta}_B{Z}_F{B}_S-\left[{d}_{CO}(T)+\psi \right]{B}_C+{d}_{OC}(T){B}_O; $$

1c $$ \frac{d{B}_O}{d\tau }={g}_O\left({B}_S,{B}_C,{B}_O;T\right){B}_O+{d}_{CO}(T){B}_C-\left[{d}_{OC}(T)+\psi \right]{B}_O; $$

1d $$ \frac{d{S}_S}{d\tau }={f}_B\left(\tau, T\right){B}_S+\varphi\;{f}_B\left(\tau, T\right){B}_C; $$

1e $$ \frac{d{S}_I}{d\tau }=\left(1-\varphi \right){f}_B\left(\tau, T\right){B}_C; $$

1f $$ \frac{d{F}_S}{d\tau }=-{\mu}_F{F}_S-{\beta}_F{Z}_B{F}_S+\zeta\;{F}_C; $$

1g $$ \frac{d{F}_E}{d\tau }={\beta}_F{Z}_B{F}_S-\left[{\mu}_F+h\;(T)\right]{F}_E; $$

1h $$ \frac{d{F}_I}{d\tau }=\left(1-\varepsilon \right)h\;(T){F}_E-\left[{\mu}_F+a\;(T)+\gamma \right]{F}_I; $$

1i $$ \frac{d{F}_C}{d\tau }=\varepsilon\;h(T){F}_E+\gamma\;{F}_I-\left({\mu}_F+\zeta \right){F}_C; $$

1j $$ \frac{d{Z}_B}{d\tau }={\pi}_B{B}_O-{\mu}_Z{Z}_B; $$

1k $$ \frac{d{Z}_F}{d\tau }={\pi}_F\left({F}_I+\kappa\;{F}_C\right)-{\mu}_Z{Z}_F, $$

where the dependence of the parameters on temperature *T* and time *τ* has been explicitly expressed.

The first terms in the right-hand sides of Eqs. (1a), (1b) and (1c) express the growth of the bryozoan biomass, which is assumed to be logistic-like. Thus we set

2 $$ {g}_X\left({B}_S,{B}_C,{B}_O;T\right)={r}_X(T)\left[1-\rho \left({B}_S+{B}_C+{B}_O\right)\right], $$

where *r*_*X*_(*T*) and *ρ* are the baseline instantaneous growth rate of class *X* = {*S*, *C*, *O*} and the inverse of the carrying capacity of the bryozoan population, respectively. We assume that susceptible and covertly infected bryozoans have the same baseline growth rate *r*_*S*_ = *r*_*C*_ = *r*, whereas *r*_*O*_ << *r* since the growth of overtly infected bryozoans is strongly impaired [36]. Both *r* and *r*_*O*_ are assumed to be monotonically increasing functions of temperature, as experimental evidence suggests [17]. The term *β*_*B*_*B*_*S*_*Z*_*F*_ in Eqs. (1a) and (1b) represents the flux of bryozoan biomass from the susceptible to the covertly infected class, with *β*_*B*_ being the exposure rate of bryozoans to fish-released spores. The transition from covert to overt infection is assumed to occur at a rate *d*_*CO*_ [see Eqs. (1b) and (1c)], which is defined as the inverse of the mean time necessary for the development of the overt stage of infection in a previously covertly infected bryozoan unit. The rate of transition from overt to covert infection is expressed by *d*_*OC*_. Both parameters are temperature-dependent: in particular *d*_*CO*_ increases with increasing temperature, while *d*_*OC*_ decreases [18]. As previously stated, the development of an overt infection, which poses a high energetic cost to the bryozoan colony, also depends on host conditions and food availability. However, as a first approximation, temperature is considered as the only determinant of change of the two transition rates. Infected bryozoans can possibly clear the infection and become again susceptible at a rate *ψ* [Eqs. (1a), (1b) and (1c)]. The production of statoblasts is only achieved by susceptible and covertly infected bryozoans, whereas overtly infected colonies do not produce statoblasts. In particular, susceptible bryozoans produce uninfected statoblasts *S*_*S*_ [Eq. (1d)] at a rate *f*_*B*_ that is assumed to depend on both temperature and time; specifically, the release of statoblasts is enhanced towards the end of the season. Seemingly, uninfected bryozoans have been observed while producing infected statoblasts as well. However, Abd-Elfattah et al. [37] argued that their observation could be interpreted as a sign of a recent loss of infection. Instead, covertly infected bryozoans can produce both infected *S*_*I*_ [Eq. (1e)] and uninfected statoblasts [37]. We term *φ* the probability that infected bryozoans produce uninfected statoblasts. The total statoblast production rate of infected bryozoans is assumed to be equal to that of susceptible ones. *T. bryosalmonae* spores [Eq. (1j)] are produced by overtly infected bryozoans at a constant rate *π*_*B*_. A constant mortality rate for spores (*μ*_*Z*_, i.e. the inverse of the time span during which spores are viable) is also accounted for.

As for the fish population, no recruitment or immigration are considered to take place during the warm season. Therefore, the abundance of susceptible fish [Eq. (1f)] decays monotonically throughout the season, due to natural mortality (at a rate *μ*_*F*_, defined as the inverse of the average lifespan of a fish) and infection from bryozoan-released spores *Z*_*B*_. The term *β*_*F*_*F*_*S*_*Z*_*B*_ in Eqs. (1f) and (1g) represents the flux of fish from the susceptible to the exposed compartment, with *β*_*F*_ being the exposure rate of fish to bryozoan-released spores. Exposed fish have already contracted the disease but are not yet infective. The decrease in the abundance of these fish, besides natural mortality, is ruled by the temperature-dependent rate *h* [Eq. (1g)]; namely, 1/*h* is the average time for the development of the disease in fish. The parameter *h* increases with increasing temperature [48]. We assume that a fraction *ε* of fish exiting from the exposed class does not show clinical symptoms and is not subject to PKD-related mortality, thus directly entering the carrier class. The remaining part (1 − *ε*) becomes infected. The abundance of infected fish [Eq. (1h)] decreases because of both natural and PKD-caused mortality. The latter is assumed to occur at a rate *a* that is positively correlated with temperature [14, 15]. In addition, infected fish can enter the carrier compartment at a rate *γ* [Eq. (1i)], which corresponds to the inverse of the average duration of the acute phase of the infection. Fish infected by the parasite that did not die in the first year reportedly continue to release infective spores for a period up to two years [43] and no evidence of infection clearing has been observed. However, field and experimental observations on the duration of this carrier stage are still scarce and further experiments are underway (Strepparava, personal communication). Therefore we explore the possibility that carrier fish may recover by introducing a recovery rate *ζ*. Evidence of possible immunity is scant (e.g. Foott & Hedrick observed immunity to second infection in rainbow trout [49]), thus, as a safe assumption, recovered fish are assumed to enter the susceptible compartment again [Eq. (1f)]. Spores [Eq. (1k)] are released by both infected and carrier fish at rates *π*_*F*_ and *κ π*_*F*_, respectively, with *κ* being an appropriate coefficient ranging from 0 to 1. Spore decay is accounted for through the parameter *μ*_*Z*_.

Since the dynamics of disease transmission between bryozoan and fish are driven by the product between the concentration of spores (*Z*_*B*_ and *Z*_*F*_) and the rates (*β*_*F*_ and *β*_*B*_) at which susceptible organisms are actually exposed to the infectious agents, we can introduce two new state variables *Z*_B_^*^ = *β*_*F*_*Z*_*B*_ and *Z*_F_^*^ = *β*_*B*_*Z*_*F*_. These new quantities are termed *equivalent spores*: namely, *Z*_F_^*^ (*Z*_B_^*^) [T^−1^] is the concentration of spores needed to infect a unit concentration of susceptible bryozoan biomass (a single susceptible fish in a unit water volume) per unit time. With this definition, the exposure rates *β*_*F*_ and *β*_*B*_ can be discarded and two synthetic contamination rates *π*_B_^*^ = *β*_*F*_*π*_*B*_ [L^3^ M^-1^ T^-2^] and *π*_F_^*^ = *β*_*B*_*π*_*F*_ [L^3^ T^-2^] are introduced. Note that both exposure and contamination rates as defined in model (1) are hardly measurable and would most likely need to be calibrated by contrasting model simulations against experimental or field data. The new parameter definitions thus reduce the number of parameters of the model and make the comparison between data and model predictions easier and more robust. The new set of equations accounting for the rescaled state variables therefore reads:

3a $$ \frac{d{B}_S}{d\tau }={g}_S\left({B}_S,{B}_C,{B}_O;T\right){B}_S-{Z}_F^{*}{B}_S+\psi \left({B}_C+{B}_O\right); $$

3b $$ \frac{d{B}_C}{d\tau }={g}_C\left({B}_S,{B}_C,{B}_O;T\right){B}_C+{Z}_F^{*}{B}_S-\left[{d}_{CO}(T)+\psi \right]{B}_C+{d}_{OC}(T){B}_O; $$

3c $$ \frac{d{F}_S}{d\tau }=-{\mu}_F{F}_S-{Z}_B^{*}{F}_S+\zeta {F}_C; $$

3d $$ \frac{d{F}_E}{d\tau }={Z}_B^{*}{F}_S-\left[{\mu}_F+h\;(T)\right]{F}_E; $$

3e $$ \frac{d{Z}_B^{*}}{d\tau }={\pi}_B^{*}{B}_O-{\mu}_Z{Z}_B^{*}; $$

3f $$ \frac{d{Z}_F^{*}}{d\tau }={\pi}_F^{*}\left({F}_I+\kappa\;{F}_C\right)-{\mu}_Z{Z}_F^{*}. $$

These equations, coupled with Eqs. (1c), (1d), (1e) (1h) and (1i) constitute the within-season model hereafter applied.

#### Between-season model

The following difference equation system relates the state of the model variables at the end of a season (*y*; *τ*_1_) with that at the beginning of the following season (*y* + 1; *τ*_0_).

4a $$ {B}_S\left(y+1;{\tau}_0\right)={\sigma}_B{B}_S\left(y;{\tau}_1\right)+\nu\;{S}_S\left(y;{\tau}_1\right); $$

4b $$ {B}_C\left(y+1;{\tau}_0\right)={\sigma}_B{B}_C\left(y;{\tau}_1\right)+{\sigma}_O{B}_O\left(y;{\tau}_1\right)+\nu\;{S}_I\left(y;{\tau}_1\right); $$

4c $$ {B}_O\left(y+1;{\tau}_0\right)=0; $$

4d $$ {S}_S\left(y+1;{\tau}_0\right)=0; $$

4e $$ {S}_I\left(y+1;{\tau}_0\right)=0; $$

4f $$ {F}_S\left(y+1;{\tau}_0\right)={\sigma}_F{F}_S\left(y;{\tau}_1\right)+{f}_F\left({F}_S\left(y;{\tau}_1\right),{F}_E\left(y;{\tau}_1\right),{F}_I\left(y;{\tau}_1\right),{F}_C\left(y;{\tau}_1\right)\right); $$

4g $$ {F}_E\left(y+1;{\tau}_0\right)=0; $$

4h $$ {F}_I\left(y+1;{\tau}_0\right)=0; $$

4i $$ {F}_C\left(y+1;{\tau}_0\right)={\sigma}_F\left[{p}_E{F}_E\left(y;{\tau}_1\right)+{p}_I{F}_I\left(y;{\tau}_1\right)+{F}_C\left(y;{\tau}_1\right)\right]; $$

4j $$ {Z}_B\left(y+1;{\tau}_0\right)=0; $$

4k $$ {Z}_F\left(y+1;{\tau}_0\right)=0. $$

At the beginning of a new season (*y* + 1; *τ*_0_), no overtly infected bryozoans are present [Eq. (4c)]; the same condition applies for statoblasts [Eqs. (4d) and (4e)] and spores [Eqs. (4j) and (4k)]. Statoblasts surviving for more than one year are here neglected for the sake of simplicity. The biomass of susceptible bryozoans [Eq. (4a)] is composed of a fraction *σ*_*B*_ of the susceptible bryozoan biomass at the end of the previous season (*y*; *τ*_1_) that managed to survive over winter, and of newly hatched colonies from the uninfected statoblasts released during the previous season. The parameter *ν* is defined as the mean amount of bryozoan biomass produced by a single statoblast.

The population of covertly infected bryozoan at the beginning of a new season [Eq. (4b)] is given by the sum of three contributions: the fraction of the covertly infected biomass at the end of the previous season that survives over winter (with probability *σ*_*B*_), the fraction of overtly infected bryozoans at time (*y*; *τ*_1_) with survival probability *σ*_*O*_, the newly established colonies generated by infected statoblasts, according to the parameter *ν*.

As for fish, we assume that a fraction *σ*_*F*_ of susceptible and carrier organisms at the end of the previous season survives over winter [Eqs. (4f) and (4i)]. Exposed and infected fish either die or enter the carrier class. This is modelled by computing additional coefficients *p*_*E*_, *p*_*I*_ accounting for natural and PKD-induced deaths:

5 $$ {p}_I=\frac{\gamma }{\mu_F+\widehat{a}+\gamma };\kern2em {p}_E=\frac{\widehat{h}}{\mu_F+\widehat{h}}\;\left[\varepsilon +\left(1-\varepsilon \right)\frac{\gamma }{\mu_F+\widehat{a}+\gamma}\right]. $$

Specifically, *p*_*E*_ (*p*_*I*_) is the probability that an exposed (infected) fish survives and enters the carrier class in the first period of the winter season. In Eq. (5) *â* and *ĥ* are the rates of PKD-caused mortality and disease development averaged over the first period of the winter season, respectively. For the sake of simplicity, we assume $\widehat{a}=a\;\left(\widehat{T}\right)$ and $\widehat{h}=h\;\left(\widehat{T}\right)$, with $\widehat{T}$ being a representative value of temperature over the considered period.

Fish reproduction occurs during winter; we assume that the amount of newborn uninfected fish (termed *f*_*F*_) depends on the total fish population at (*y*; *τ*_1_):

6 $$ {f}_F\left(\tilde{F}\right)=\eta\;\tilde{F} \exp \left(-\xi\;\tilde{F}\right), $$

where $\tilde{F}={F}_S\left(y;{\tau}_1\right)+{p}_E{F}_E\left(y;{\tau}_1\right)+{p}_I{F}_I\left(y;{\tau}_1\right)+{F}_C\left(y;{\tau}_1\right)$. Eq. (6) assumes a Ricker growth model [46]. The parameter *η* is the baseline fertility rate, i.e. the average number of offspring produced by a single fish when the total fish population size is low, while *ξ* determines the strength of density dependence. For the aforementioned reasons, at the beginning of a season there are neither exposed nor infected fish [Eqs. (4g) and (4h)]. Table 2 summarizes all parameters of systems (1) and (4).

**Table 2:** List of parameters

Parameter	Definition	Dimension
Bryozoans		
Constant		
*ρ*	Inverse of carrying capacity	[M^-1^L^-3^]
*β*_*B*_	Exposure rate	[L^3^T^-1^]
*φ*	Fraction of *B*_*C*_ generating uninfected statoblasts	[−]
*ψ*	Recovery rate	[T^-1^]
*π*_*B*_	Rate of contamination operated by *B*_*O*_	[M^-1^T^-1^]
*σ*_*B*_	Probability of survival over winter for *B*_*S*_, *B*_*C*_	[−]
*σ*_*O*_	Probability of survival over winter for *B*_*O*_	[−]
*ν*	Biomass generated by one statoblast	[M]
Temperature-dependent		
*r*	Baseline growth rate of *B*_*S*_, *B*_*C*_	[T^-1^]
*r*_*O*_	Baseline growth rate of *B*_*O*_	[T^-1^]
*d*_*CO*_	Rate of covert-to-overt transition	[T^-1^]
*d*_*OC*_	Rate of overt-to-covert transition	[T^-1^]
Time and temperature-dependent		
*f*_*B*_	Statoblast production rate	[M^-1^T^-1^]
Fish		
Constant		
*μ*_*F*_	Natural mortality rate	[T^-1^]
*β*_*F*_	Exposure rate	[L^3^T^-1^]
*ε*	Fraction of acute infections	[−]
*γ*	Rate of recovery from acute infection	[T^-1^]
*ζ*	Rate of complete recovery	[T^-1^]
*π*_*F*_	Rate of contamination operated by *F*_*I*_	[T^-1^]
*κ*	Relative rate of contamination operated by *F*_*C*_	[−]
*σ*_*F*_	Probability of survival over winter	[−]
*η*	Baseline reproduction rate	[−]
*ξ*	Strength of density dependence	[L^-3^]
Temperature-dependent		
*h*	Rate of development of the disease	[T^-1^]
*a*	PKD-caused mortality rate	[T^-1^]
Parasite		
*μ*_*Z*_	Spore decay rate	[T^-1^]
Rescaled parameters		
${\pi}_B^{*}={\beta}_F{\pi}_B$	Synthetic rate of contamination operated by *B*_*O*_	[L^3^M^-1^T^-2^]
${\pi}_F^{*}={\beta}_B{\pi}_F$	Synthetic rate of contamination operated by *F*_*I*_	[L^3^T^-2^]

### Model simulation

**Fig. 2:**
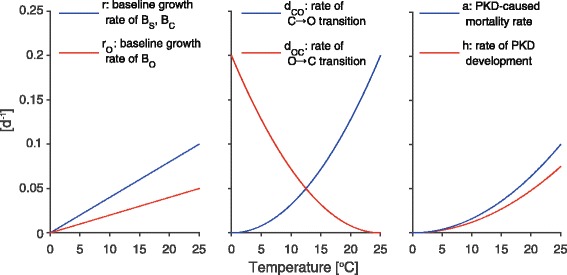
Functional forms for temperature-dependent parameters. The maximum values of these parameters are set to match the upper limits proposed in Table 3. Furthermore, we assume ${f}_B=0.005\kern0.22em T\kern0.28em \left[{}^{\mathrm{o}}\mathrm{C}\right]+0.00025\kern0.22em \tau \kern0.28em \left[\mathrm{d}\right]$, where *τ* is the time elapsed since January 1

In order to understand possible patterns of disease dynamics, we ran model simulations. The feasible range of several model parameters was estimated based on literature values and experts’ knowledge. Reasonable values were assumed for the remaining ones. Reference parameter values and feasible ranges are reported in Table 3. In the absence of detailed information about recovery mechanisms for fish and bryozoans, we assumed slow recovery rates as reference values (average recovery time equal to half of the lifetime for fish and half of the yearly proliferation period for bryozoan) and explored a wide range of possible values, including the absence of infection-clearing mechanisms (*ψ* = *ζ* = 0). As for temperature-dependent parameters, linear (*r*, *r*_*O*_) or parabolic (*d*_*CO*_, *d*_*OC*_, *h*, *a*) relationships have been assumed; a superlinear dependence on *T* for the latter parameters was deduced from literature review (see references in Table 3). The functional forms used are illustrated in Fig. 2. Note that, for these parameters, the corresponding reference values are obtained from the relationships of Fig. 2 by assuming *T* = 15 °C. For the numerical simulations, we use a time series of stream water temperature measured in River Langeten, Switzerland, in the period 2002–2013 (data provided by the Swiss Federal Office for the Environment - FOEN).

**Table 3:** Parameter ranges and reference values

Parameter	Range	Reference value	Unit	Source
*r*	0.01 − 0.1	0.06	d^-1^	[17]
*r*_*O*_	0.005 − 0.05	0.03	d^-1^	[17]
*ψ*		0.01	d^-1^	
*ρ* ^− 1^		20	gm^-3^	
*d*_*OC*_	0.02 − 0.2	0.032	d^-1^	[18]
*d*_*CO*_	0.02 − 0.2	0.072	d^-1^	[18]
*a*	0.015 − 0.1	0.036	d^-1^	[14, 15]
*h*	0.01 − 0.075	0.027	d^-1^	[6, 19, 48]
*γ*	0.01 − 0.05	0.02	d^-1^	[43]
*ζ*		0.001	d^-1^	[43]
*ε*		0.1	–	
*π*_B_^*^		0.005	m^3^ g^-1^ d^-2^	
*π*_F_^*^		0.1	m^3^ d^-2^	
*κ*		0.2	–	
*μ*_*F*_^−1^	~ 5 years	2000	d	
*μ*_*Z*_^−1^	≤ 24 h	0.75	d	[19, 62]
*σ*_*B*_	0 − 0.3	0.1	–	
*σ*_*O*_	0 − 0.1	0.05	–	
*σ*_*F*_		0.9	–	
*η*		1	–	
*ξ* ^− 1^	≤ 1.5	0.5	m^-3^	[63]
*ν*		0.035	g	
*f*_*B*_		0.1	g^-1^ d^-1^	
*φ*	0.55 − 0.8	0.7	–	[37]
$\widehat{T}$		10	°C

### Analysis of the within-season model

Parasite invasion in a previously PKD-free system most likely occurs during the warm season. For this reason, we first focus our analysis on the continuous, within-season model and investigate the conditions under which the introduction of the parasite in a fully susceptible population of fish and bryozoans (say through the introduction of spores, infected fish or infected bryozoans) leads to an outbreak of PKD. To this end, we analyze the linear stability of the disease-free equilibrium (DFE) in a simplified model that disregards population dynamics of both fish and bryozoans (i.e. both population sizes are treated as model parameters). Technical details are provided in Additional file 1. The instability of the within-season DFE would indicate long-term persistence of the parasite only if model parameters were kept constant at the value used for the computation of the stability criterion. However, in our system, population dynamics and water temperature variations constantly affect parameters values. Also, the alternation of warm and cold seasons, each endowed with different eco-epidemiological processes, de facto prevents a stable, nontrivial steady-state from being established in the system. Therefore, the instability of the within-season DFE can be seen as a useful indicator only of the short-term invasibility of the system. To investigate long-term parasite persistence and the establishment of endemic transmission conditions, a more complex analysis that accounts for both seasonal forcing and intra- and inter-seasonal population and epidemiological dynamics is required, as illustrated in the following section.

### Stability analysis of the full model

As the seasonal cycle of water temperature critically controls PKD transmission and bryozoan population dynamics, we study the stability of the disease-free trajectory (DFT), a succession of system states in which the disease is not present and endemic transmission is not possible. To achieve this aim, we define a Poincaré map coupling between-season update with the within-season dynamics, with the latter being described through an integral operator derived from Floquet theory [50–52], a mathematical framework that allows the analysis of systems of periodically forced differential equations. As this theory assumes periodic forcing, the time evolution of water temperature is approximated via a sinusoidal signal with yearly period.

#### Disease-free trajectory

The DFT is defined as a discrete-continuous trajectory describing the yearly periodic evolution of biomass or abundances for the uninfected classes (*B*_*S*_, *S*_*S*_, *F*_*S*_) in the absence of PKD. Therefore, we first focus on a reduced system where only susceptible compartments are taken into account. Let **x**_S_(*y*; *τ*) = {*B*_*S*_(*y*; *τ*); *S*_*S*_(*y*; *τ*); *F*_*S*_(*y*; *τ*)} be the state of this system at season *y* and time *τ*. The time-evolution of this system can be represented as follows:

$$ {\mathbf{x}}_{\mathrm{S}}\left(y;{\tau}_0\right)\overset{v\left(\cdot \right)}{\to }{\mathbf{x}}_{\mathrm{S}}\left(y;{\tau}_1\right)\overset{w\left(\cdot \right)}{\to }{\mathbf{x}}_{\mathrm{S}}\left(y+1;{\tau}_0\right)\overset{v\left(\cdot \right)}{\to }{\mathbf{x}}_{\mathrm{S}}\left(y+1;{\tau}_1\right) $$

where *v*(⋅) is an operator describing the continuous-time dynamics of the system from *τ*_0_ to *τ*_1_, while *w*(⋅) is an operator describing the discrete-time update of the system between the end of season *y* and the beginning of season *y* + 1. Therefore, we obtain:

$$ {\mathbf{x}}_{\mathbf{S}}\left(y+1;{\tau}_0\right)=w\left(v\left({\mathbf{x}}_{\mathbf{S}}\left(y;{\tau}_0\right)\right)\right)=\left(w\circ v\right)\left({\mathbf{x}}_{\mathbf{S}}\left(y;{\tau}_0\right)\right), $$

where *w* ∘ *v* defines a so-called Poincaré map [53]. A fixed point of the Poincaré map is defined as an equilibrium $\overline{{\mathbf{x}}_{\mathbf{S}}}\left(y;\tau \right)$ such that $\overline{{\mathbf{x}}_{\mathbf{S}}}\left(y;{\tau}_0\right)=\overline{{\mathbf{x}}_{\mathbf{S}}}\left(y+1;{\tau}_0\right)=\left(w\circ v\right)\left(\overline{{\mathbf{x}}_{\mathbf{S}}}\left(y;{\tau}_0\right)\right)$.

The disease-free trajectory $\overline{{\mathbf{x}}_{\mathbf{df}}}\left(y;\tau \right)$ for systems (1) and (4) is defined under the hypothesis that water temperature can be approximated by a sinusoidal function of time, with period equal to one year. We therefore obtain

7 $$ \overline{{\mathbf{x}}_{\mathbf{S}}}\left(y;\tau \right)=\left\{\frac{B_{S,0}}{\rho {B}_{S,0}+\left(1-\rho {B}_{S,0}\right) \exp \left(-R\left(\tau \right)\right)},0,{F}_{S,0} \exp \left(-{\mu}_F\left(\tau -{\tau}_0\right)\right)\right\}, $$

where

$$ R\left(\tau \right)={\displaystyle {\int}_{\tau_0}^{\tau }}rdt $$

and *B*_*S*,0_ (*F*_*S*,0_) is the susceptible bryozoan (fish) population at *τ*_0_ along the DFT. Note in fact that $\overline{{\mathbf{x}}_{\mathbf{S}}}\left({\tau}_0\right)=\left\{{B}_{S,0},,,0,{F}_{S,0}\right\}$. *B*_*S*,0_ is the solution of the following trascendental equation:

$$ 1-\frac{\sigma_B}{\rho {B}_{S,0}+\left(1-\rho {B}_{S,0}\right) \exp \left(-R\left({\tau}_1\right)\right)}-{\displaystyle {\int}_{\tau_0}^{\tau_1}}\frac{\nu {f}_B}{\rho {B}_{S,0}+\left(1-\rho {B}_{S,0}\right) \exp \left(-R\left(\tau \right)\right)}d\tau =0, $$

The value of *B*_*S*,0_ can be obtained numerically for a given set of parameters. Instead, an analytical expression can be found for *F*_*S*,0_:

8 $$ {F}_{S,0}={\xi}^{-1} \ln \left[\frac{\eta }{ \exp \left({\mu}_F\left({\tau}_1-{\tau}_0\right)\right)-{\sigma}_F}\right] \exp \left({\mu}_F\left({\tau}_1-{\tau}_0\right)\right). $$

Therefore, $\overline{{\mathbf{x}}_{\mathbf{df}}}\left(y;\tau \right)$ is the trajectory such that its susceptible components are equal to (7), while its infected components **x**_**I**_ = {*B*_*C*_; *B*_*O*_; *S*_*I*_; *F*_*E*_; *F*_*I*_; *F*_*C*_; *Z*_B_^*^; *Z*_F_^*^} are null. Note that Eq. (8) requires *η* > *η*_*min*_ = exp(*μ*_*F*_(*τ*_1_ − *τ*_0_)) − *σ*_*F*_ in order to avoid extinction of fish. For the reference parameter set of Table 3, one gathers *η*_*min*_ ≈ 0.205.

#### Stability of the disease-free trajectory

In order to study the stability of the DFT $\overline{{\mathbf{x}}_{\mathbf{df}}}\left(y;\tau \right)$ of model (1) and (4) with respect to small perturbations, we refer to the reduced system where only infected compartments are considered. Hence, **x**_**I**_(*y*; *τ*) denotes the state of such a system, whose time evolution is represented as follows:

9 $$ {\mathbf{x}}_{\mathbf{I}}\left(y;{\tau}_0\right)\overset{\mathbf{V}\left(\cdot \right)}{\to }{\mathbf{x}}_{\mathbf{I}}\left(y;{\tau}_1\right)\overset{\mathbf{W}\left(\cdot \right)}{\to }{\mathbf{x}}_{\mathbf{I}}\left(y+1;{\tau}_0\right)\overset{\mathbf{V}\left(\cdot \right)}{\to }{\mathbf{x}}_{\mathbf{I}}\left(y+1;{\tau}_1\right). $$

**V** and **W** are operators describing the evolution of the linearized system either within or between seasons. Note that the fixed point for this system is the null vector.

The propagation matrix **V** is given by the solution of the following matrix ODE system:

10 $$ \frac{d\;\mathbf{Z}}{d\tau }=\mathbf{A}\left(\tau \right)\mathbf{Z}, $$

where **A**(τ) is a matrix obtained from the Jacobian of the within-season system (1), evaluated along the trajectory $\overline{{\mathbf{x}}_{\mathbf{df}}}\left(y;\tau \right)$, where all rows and columns related to the susceptible compartments have been removed:

$$ \mathbf{A}\left(\tau \right)=\left[\begin{array}{cccccccc}\hfill {A}_{11}\hfill & \hfill {d}_{OC}\hfill & \hfill 0\hfill & \hfill 0\hfill & \hfill 0\hfill & \hfill 0\hfill & \hfill 0\hfill & \hfill {B}_S\hfill \\ {}\hfill {d}_{CO}\hfill & \hfill {A}_{22}\hfill & \hfill 0\hfill & \hfill 0\hfill & \hfill 0\hfill & \hfill 0\hfill & \hfill 0\hfill & \hfill 0\hfill \\ {}\hfill \left(1-\varphi \right){f}_B\hfill & \hfill 0\hfill & \hfill 0\hfill & \hfill 0\hfill & \hfill 0\hfill & \hfill 0\hfill & \hfill 0\hfill & \hfill 0\hfill \\ {}\hfill 0\hfill & \hfill 0\hfill & \hfill 0\hfill & \hfill -{\mu}_F-h\hfill & \hfill 0\hfill & \hfill 0\hfill & \hfill {F}_S\hfill & \hfill 0\hfill \\ {}\hfill 0\hfill & \hfill 0\hfill & \hfill 0\hfill & \hfill \left(1-\varepsilon \right)h\hfill & \hfill {A}_{55}\hfill & \hfill 0\hfill & \hfill 0\hfill & \hfill 0\hfill \\ {}\hfill 0\hfill & \hfill 0\hfill & \hfill 0\hfill & \hfill \varepsilon\;h\hfill & \hfill \gamma \hfill & \hfill -{\mu}_F-\zeta \hfill & \hfill 0\hfill & \hfill 0\hfill \\ {}\hfill 0\hfill & \hfill {\pi}_B^{*}\hfill & \hfill 0\hfill & \hfill 0\hfill & \hfill 0\hfill & \hfill 0\hfill & \hfill -{\mu}_Z\hfill & \hfill 0\hfill \\ {}\hfill 0\hfill & \hfill 0\hfill & \hfill 0\hfill & \hfill 0\hfill & \hfill {\pi}_F^{*}\hfill & \hfill \kappa\;{\pi}_F^{*}\hfill & \hfill 0\hfill & \hfill -{\mu}_Z\hfill \\ {}\hfill \hfill & \hfill \hfill & \hfill \hfill & \hfill \hfill & \hfill \hfill & \hfill \hfill & \hfill \hfill & \hfill \hfill \end{array}\right], $$

where *A*_11_ = *r*(1 − *ρ B*_*s*_) − (*d*_*CO*_ + *ψ*), *A*_22_ = *r*_*O*_(1 − *ρ B*_*s*_) − (*d*_*OC*_ + *ψ*), *A*_55_ = − *μ*_*F*_ − *a* − *γ*. Equation (10) must be integrated from *τ*_0_ to *τ*_1_, with initial condition **Z**(*τ*_0_) = **I** (identity matrix). We thus have **V** = **Z**(*τ*_1_).

Matrix **W** can be obtained from the Jacobian matrix of the between-season system (4), by disregarding all rows and columns related to susceptible compartments. **W** is a sparse matrix of order 8 with the following non-null entries: *W*_11_ = *σ*_*B*_, *W*_12_ = *σ*_*O*_, *W*_13_ = *ν*, *W*_64_ = *p*_*E*_*σ*_*F*_, *W*_65_ = *p*_*I*_*σ*_*F*_, *W*_66_ = *σ*_*F*_.

The stability of the disease-free trajectory of system (1) and (4) corresponds to the stability of the fixed point **x**_**I**_ = **0** of the Poincaré map defined in (9), i.e. **x**_**I**_(*y* + 1; *τ*_1_) = **VW x**_**I**_(*y*; *τ*_1_). Therefore, the necessary and sufficient condition for the exponential instability of the DFT reads

11 $$ {\lambda}_{max}= \max \left|\lambda \left(\mathbf{V}\mathbf{W}\right)\right|>1, $$

where *λ* are the eigenvalues of matrix **VW**. When condition (11) is met, the parasite can invade the system. We therefore focus our analysis on *λ*_*max*_, i.e. the maximum modulus of the eigenvalues of **VW**. For a given set of parameters, the corresponding value of *λ*_*max*_ can be computed numerically.

### Sensitivity analyses

**Fig. 3:**
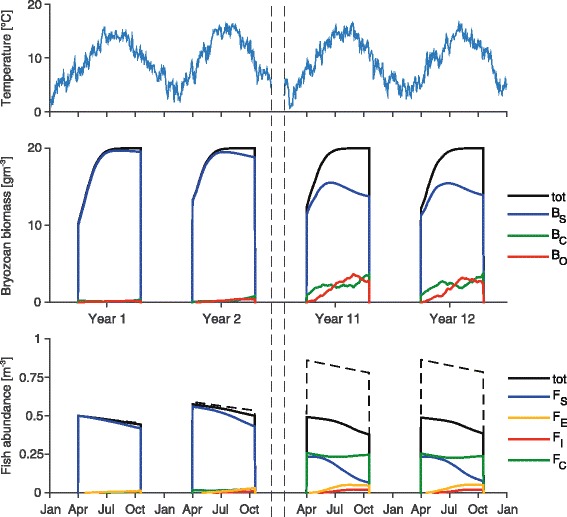
Model simulation. Evolution of the state variables of model (1) and (4) in a 12-years-long simulation. Seasons start on April 1 and last for 200 days. Black solid lines represent the time evolution of the overall bryozoan and fish populations; the dashed line indicates fish abundance if PKD is absent. Initial condition: *B*_*S*_ = 0.495*ρ* ^-1^, *B*_*C*_ = 0.005*ρ* ^-1^, *F*_*S*_ = 0.99*ξ* ^-1^, *F*_*C*_ = 0.01*ξ* ^-1^; other state variables are null. Temperature-dependent parameters are taken from Fig. 2; other parameters are set to their reference values (reported in Table 3). The water temperature time series used for the simulation is reported in the top subplot

To understand how model parameters affect parasite invasibility and long-term PKD impact, we performed sensitivity analyses. Specifically, we investigate the effect of parameters on the value of *λ*_*max*_ and on the PKD-induced fish loss, here estimated as the percentage of fish at the end of the season with respect to the fish population size if the disease were absent. Computations are run by varying two focus parameters at a time while keeping the others at their reference value, as specified in Table 3. Temperature-dependent parameters are expressed via the functional forms of Fig. 2. The effect of these parameters is explored by varying their value at 15 °C, while keeping their minimum value at 0 °C (at 25 °C for *d*_*OC*_) constant. For parabolic functional forms, the null derivative at their minimum is also kept constant. The sinusoidal signal of water temperature is derived from the time series shown in the top panel of Fig. 3. Seasons are assumed to start on April 1 and last for 200 days. The effect of water temperature is also investigated by varying both the yearly mean value and the relative mid-amplitude of the sinusoidal signal, while using the relationships of Fig. 2.

## Results

### Model simulation

Figure 3 shows an example of model simulation where it is assumed that at the beginning of the first season 1 % of the bryozoans are covertly infected and 1 % of the fish belong to the carrier class. The remaining fractions of the host populations are susceptible. This model setting mimics the invasion of the parasite in a fully susceptible system. The total bryozoan population tends to reach the carrying capacity *ρ*^-1^ towards the end of the season. Overt stages of infection are absent in early spring but peaks are observed in summer. The irregular shape of the curves for *B*_*C*_ and *B*_*O*_ mirrors the variability of water temperature. Regarding fish, peaks of infection are observed during summer. After a few years, prevalence reaches about 80 % at the end of the summer, where few susceptible fish are present and most of the survived individuals belong to the carrier class. This is in agreement with the fact that, although young-of-the-year fish surviving the infection are not likely to develop clinical PKD in the following year, the parasite can remain viable in the host for several seasons after initial exposure [16, 43, 54]. In this model simulation, some 28 % of the fish population which is alive at *τ*_0_ dies during the season because of PKD. About ten years after the initial invasion, the disease becomes endemic and the seasonal state variables’ trajectories remain virtually unchanged. Overall, the model reproduces patterns of disease spread in bryozoan and fish populations which are in good qualitative agreement with evidence from the literature and field observations (see [19]).

### Analysis of the within-season model

**Fig. 4:**
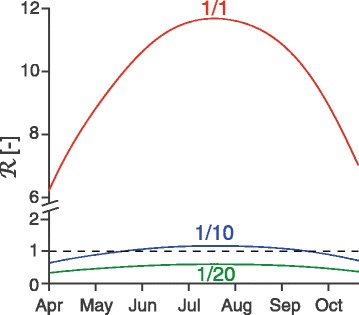
Short-term risk of invasibility. Reproductive number $\mathcal{R}$ as a function of time. Water temperature is approximated as a sinusoidal signal (as for the computation of the DFT) and temperature-dependent parameters are taken as in Fig. 2. *F*_*S*_(*τ*) and *B*_*S*_(*τ*) follow the DFT. The red line refers to the reference parameter set reported in Table 3. The blue and the green lines show the effect of reducing one of the transmission parameters (*π*_B_^*^ or *π*_F_^*^) or the fish population size *F*_*S*_ by a factor 10 and 20, respectively

The analysis of the within-season model (Additional file 1) reveals two types of instability of the DFE. The first is related to the sign of the quantity

$$ \mathcal{T}={\psi}^2+\left[{d}_{CO}+{d}_{OC}-\left(r+{r}_O\right)\left(1-\rho {B}_S\right)\right]\psi -\left(1-\rho {B}_S\right)\left[{r}_O{d}_{CO}+r{d}_{OC}+r{r}_O\left(1-\rho {B}_S\right)\right], $$

which only depends on parameters regarding the bryozoan sub-model (note that the biomass of susceptible bryozoan *B*_*S*_ is assumed as a model parameter for this analysis). When $\mathcal{T}<0$, the DFE is unstable and the parasite can spread in the bryozoan population even in the absence of the fish host. Notably, if bryozoan cannot recover (*ψ* = 0), $\mathcal{T}$ is always negative, as long as the bryozoan biomass is lower than the carrying capacity. When $\mathcal{T}>0$, the parasite needs to cycle between the two hosts to possibly invade the system and the instability of the DFE occurs when the reproductive number

$$ \mathcal{R}=\frac{B_S{F}_S{d}_{CO}h{\pi}_B^{*}{\pi}_F^{*}\left[\varepsilon \kappa a+\kappa \gamma +\left(1-\varepsilon \right)\zeta +\left(1+\varepsilon \kappa -\varepsilon \right){\mu}_F\right]}{\mathcal{T}\left(h+{\mu}_F\right)\left(a+\gamma +{\mu}_F\right)\left(\zeta +{\mu}_F\right){\mu}_Z^2} $$

is greater than unity. Starting from the reference parameter set, $\mathcal{R}$ increases as parameters *F*_*S*_, *d*_*CO*_, *h*, *π*_B_^*^, *π*_F_^*^, increase, and decreases as parameters *ρB*_*S*_, *d*_*OC*_, *a*, *ψ* increase (see Additional file 1). Figure 4 illustrates how $\mathcal{R}$ evolves during a season, as temperature affects parameter values, and the population sizes of fish and bryozoan follow the DFT. The effect of temperature dominates and maximizes the reproductive number, and the related risk of an outbreak, during the warmest period. With the reference parameter set, $\mathcal{T}>0$ and $\mathcal{R}$ > 1 throughout the season: the parasite requires the fish host to spread into the system and the DFE is always unstable. Figure 4 also shows how reducing one of the transmission parameters (*π*_B_^*^ or *π*_F_^*^) or the fish population size *F*_*S*_ by a factor 10 and 20 can curb the reproductive number below unity during the coolest periods and throughout the season, respectively.

### Sensitivity analyses of the full model

#### Parasite invasion

**Fig. 5:**
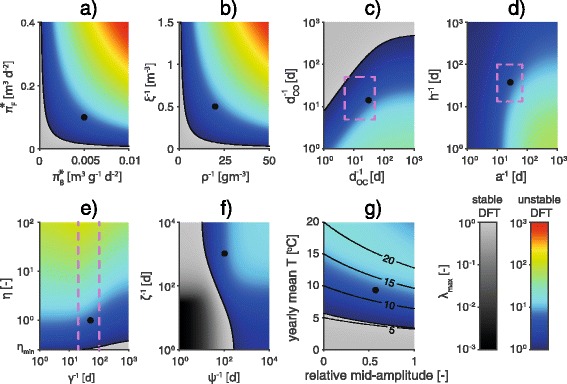
Sensitivity analysis: Parasite invasion. Values of *λ*_*max*_ as a function of model parameters *π*_B_^*^​ vs. *π*_F_^*​^ (**a**); *ρ* vs. *ξ* (**b**); *d*_*OC*_ vs. *d*_*CO*_ (**c**); *a* vs. *h* (**d**); *γ* vs. η (**e**); *ψ* vs. *ζ* (**f**); temperature (**g**). Pink dashed lines identify feasible parameter ranges. Black dots refer to the reference parameter set. For the sake of clarity, all rates are expressed as mean times (i.e. by their inverse). With regards to temperature-dependent parameters, their value at 15 °C is displayed. Black solid lines in **g** identify levels of mean temperature during the warm season

An exploration of the values of *λ*_*max*_ over wide ranges of the parameter space is reported in Fig. 5. Numerical results show high invasibility of the system (*λ*_*max*_ > 1) for wide ranges of realistic parameters. The DFT becomes stable if one of the contamination rates (*π*_B_^*^ and *π*_F_^*^, Fig. 5a) or if the characteristic sizes of bryozoan or fish populations (*ρ*^-1^ and *ξ*^-1^, Fig. 5b) are small. Recovery mechanisms (*ψ* and *ζ*, Fig. 5f) can promote the stability of the DFT: in particular, the DFT is stable if infection-clearing in bryozoans is fast enough, whereas if only *ζ* is increased, the persistence of the infection in bryozoans hinders the stability of the DFT. The DFT is predicted to be stable also if the rate of transition from overt to covert infection *d*_*OC*_ is considerably higher than the antithetic rate *d*_*CO*_ (Fig. 5c), although this circumstance occurs for unlikely values of these parameters. Finally, stability of the DFT is observed for extremely low values of the fish reproduction and recovery rates (*η* and *γ*, Fig. 5e).

When the DFT is unstable, the maximum modulus *λ*_*max*_ of the eigenvalues of matrix **VW** can be interpreted as the rate at which model trajectories depart from the disease-free trajectory. *λ*_*max*_ thus represents a measure of how fast the parasite spreads into a susceptible population. When *λ*_*max*_ is slightly greater than unity, the outbreak develops slowly and stochastic effects (e.g. due to the demographic stochasticity of fish and bryozoan infected populations), which are not accounted for in the current model formulation, may lead to the extinction of the disease. When the DFT is stable, the closer *λ*_*max*_ is to unity, the slower perturbations to the DFT fade out and the system returns to a disease-free state. Therefore, *λ*_*max*_ can also be seen as an approximate estimate of the actual risk of invasion. In this perspective, Fig. 5 provides information on the role of the model parameters in promoting the establishment of PKD in fish populations. *π*_B_^*^ and *π*_F_^*^ have analogous effects in enhancing the risk for an outbreak. *ρ*^-1^, *ξ*^-1^ and the fish baseline fertility rate *η* are also positively correlated with *λ*_*max*_. Concerning the effects of transmission and mortality rates, the highest values of *λ*_*max*_ are found when the disease development rate *h* is maximum and PKD-caused mortality *a* is minimum (Fig. 5d). This condition maximizes the number of non-fatal infection in fish, thereby causing an increase in the release of spores *Z*_*F*_. As for the thermal regime, the mean water temperature during the warm season stands as the main factor controlling *λ*_*max*_ (Fig. 5g).

#### PKD-induced fish loss

**Fig. 6:**
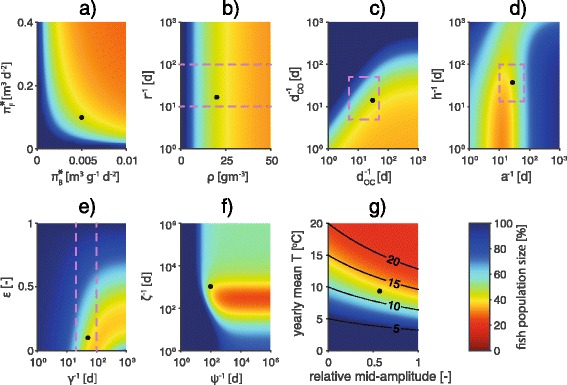
Sensitivity analysis: PKD-induced fish loss as a function of model parameters *π*_B_^*​^ vs. *π*_F_^*^​ (**a**); *ρ* vs. *r* (**b**); *d*_*OC*_ vs. *d*_*CO*_ (**c**); *a* vs. *h* (**d**); *γ* vs. *ε* (**e**); *ψ* vs. *ζ* (**f**); temperature (**g**). Simulations are run until convergence (100 seasons), with each season lasting for 200 days. Colors refer to the percentages of the population size at the end of the last season with respect to the same quantity calculated along the disease-free trajectory [computed as in Eqs. (7) and (8)]. For example, 50 % means that at the end of the last season the population size is half of the population that would have survived if the disease were absent. State variables at the beginning of the first season are set as in the model simulation of Fig. 3. Pink dashed lines identify feasible parameter ranges. Black dots refer to the reference parameter set. All rates are represented as mean times. With regards to temperature-dependent parameters, their value at 15 °C is displayed. Black solid lines in **g** identify levels of mean temperature during the warm season

Figure 6 shows the results of the sensitivity analysis of PKD impact on fish population size. The residual population size is larger (i.e. PKD impact is reduced) for low values of the contamination rates and the bryozoan carrying capacity (Fig. 6a and b), whereas no sensitivity to high values of these parameters is observed. The bryozoan baseline growth rate (Fig. 6b) has no effect on PKD-induced fish loss. Fish population is preserved when *d*_*OC*_ is high and *d*_*CO*_ is low, while the opposite case does not result in a severe population decay (Fig. 6c). Expectedly, there is a positive correlation between PKD-induced fish loss and PKD-induced mortality rate *a* (Fig. 6d). However, if *a* is extremely high, the immediate death of the host limits the transmission of the disease and thus its impact, a common feature of epidemiological models. PKD impact is enhanced by the rate of disease development *h*. Fish population decay is also enhanced when both *ε* and *γ* are low, as this condition maximizes the abundance of acutely infected fish (Fig. 6e); as previously pointed out, if *γ* is extremely low, this effect is balanced by the augmented relative importance of *a*, which weakens PKD impact. As the recovery time of bryozoans, *ψ*^-1^, decreases, PKD impact monotonically decreases (Fig. 6f). On the other hand, PKD impact reaches a peak for intermediate rates of fish recovery (*ζ* ≈ 0.005 d^-1^), presumably due to concomitant high level of contamination and increased availability of susceptible fish.

As expected, higher water temperatures promote higher fish mortality (Fig. 6g). In particular, PKD impact is mostly related to the mean temperature during the warm season, whereas the amplitude of the temperature sinusoidal signal plays a lesser role. For instance, assuming a relative mid-amplitude of 0.5, an increase of 5 °C (from 10 to 15 °C) in the seasonal mean water temperature would produce an overall reduction of 26 % of the total fish population.

## Discussion

According to the modelling framework developed in this work, the disease-free trajectory is found to be unstable over wide ranges of the ecological and epidemiological parameters. This property means that the introduction of the parasite in a fully susceptible community of salmonids and bryozoans would very likely lead to a PKD outbreak and to long-term parasite establishment. The ability of the parasite to cycle between covert, non-virulent, and overt, transmissive phases in the bryozoan host allows the parasite to produce large numbers of transmissive stages without compromising the susceptible host population. Similarly, long-term infections in trout are also responsible for continuous infections of naive bryozoan hosts and promote parasite persistence. This result holds for the deterministic model (1) and (4). Stochastic effects, currently not accounted for in the model formulation, could actually prevent the invasion. Our findings obviously depend on the current knowledge of the transmission modes of PKD (and on how it has been translated into mathematical terms), and the lack thereof. Indeed, the analyses of the within-season and the full models show how recovery mechanisms for both bryozoans and fish reduce the risk of outbreak and can make the disease-free system stable. However, knowledge and investigations on these critical processes are still scant. While the existence of a recovery dynamic for bryozoans is currently unknown (but see [37]), some experiments conducted on rainbow trout [55] actually revealed hints of a possible recovery from infection in salmonids. Further studies on this topic are needed to better elucidate the mechanisms underlying the persistence of *T. bryosalmonae* in fish and bryozoan communities. Overall, these results underline the reasons for the emerging status of PKD throughout Europe - the extremely successful invasion mechanism of the parasite may have facilitated its historical spread in European streams, and the current changes in climate and other environmental conditions are causing it to proliferate.

Our results highlight how both outbreak risk and the long-term persistence of the parasite are critically enhanced by warmer water temperature. The design of possible control strategies (e.g. the control of the population of one of the host or the interactions among them) should thus take into account that warmer periods pose a higher risk of a PKD outbreak. On the other hand, the effect of temperature offers the opportunity to design alternative intervention strategies aimed at controlling stream water temperature through e.g. tree shading or the selective release of cold water from upstream reservoirs. The analysis of the within-season model also shows that, for certain parameter combinations, the parasite can initially spread even in the absence of the fish host. This possible behavior is determined by the fact that the model assumes that infected bryozoan grow and that *T. bryosalmonae* can simultaneously proliferate inside them. This specific dynamics should be better scrutinized with additional experimental studies because the possibility of epidemics hosted solely by the bryozoan population has relevant implications for control strategies. It implies, for instance, that strategies focusing only on the fish population or on limiting the interactions between the two hosts might not be effective, under certain conditions, to prevent the invasion of the parasite.

Simulations and sensitivity analyses provided insights about the role and importance of the different parameters in parasite invasion and outbreak severity. In particular, the analysis of PKD-induced fish loss highlights the crucial role of the parameters that govern the transition between epidemiological classes in determining the impact of PKD outbreaks when the disease is endemic. While the carrying capacity of bryozoans and the contamination rates are the main factors controlling the stability of the disease-free trajectory, they appear less relevant in determining PKD impact in endemic settings. The underlying reason for this result is that these parameters solely control the rate at which susceptible fish are exposed to the parasite, whereas the infection develops over time scales ruled by the parameters governing the transition between epidemiological classes. A promising aspect is the fact that such parameters and their temperature dependence could be estimated in laboratory experiments by exposing hosts to the parasite and by monitoring the development of their infection status. Similar studies have already been carried out (see corresponding references in Table 3). More are needed, however, to better elucidate the role of temperature on PKD dynamics. It should be noted, in fact, that most of the available literature on PKD is based on rainbow trout (*Oncorhynchus mykiss*), which is known to be a dead-end host for *T. bryosalmonae*, as this species does not allow the completion of the parasite cycle by further infecting bryozoans [56]. Conversely, only few studies have focused on brown trout [43, 56, 57].

While it could be relevant to experimentally assess how the release of spores (by both infected fish and bryozoans) varies with temperature, the estimation of the actual value of the release rate may be less important. Indeed, the analysis of the model reveals that transmission dynamics are controlled by the product between contamination and exposure rates. The latter can hardly be estimated under field conditions as they depend on the probability of a successful encounter between viable spores and hosts. Therefore, efforts to precisely estimate contamination rates would be frustrated by the large uncertainties associated to exposure rates. The proposed rescaled model (3) features two parameters (one per host) that represent the product between contamination and exposure rates. Such parameters are key to disease dynamics, as discussed above. However, reasonable values can hardly be estimated a priori, and would need to be calibrated for each case study by contrasting model simulations with experimental or field data.

Knowledge and literature on bryozoans are rather scant compared to the vast and traditional literature on population dynamics and habitat distribution of salmonids. To improve our understanding of PKD as an emerging disease, such knowledge gap must be filled. In particular, knowledge of bryozoan habitat suitability needs be improved for an effective mapping on the risk of PKD invasion. This task could be achieved by using species distribution models [58] to relate the presence/absence of bryozoans to environmental variables (e.g. hydrological conditions, characteristics of the river-bed and banks, water quality, temperature). The few existing studies (e.g. [59]) focus on lakes, while large-scale studies of habitat suitability in streams and rivers are not available yet. Furthermore, many aspects of the relationship between infection status and bryozoan proliferation are still to be elucidated: for example, population genetic diversity of bryozoans may be linked to their ability to resist to infection; infective stages may propagate in partially infected bryozoan zooids even in the absence of further exposure to *T. bryosalmonae*.

Our results need be accompanied by an assessment of the limits of the model. It is acknowledged that young-of-the-year fish are highly likely to contract PKD when exposed to the parasite for the first time [60]. However, for the sake of simplicity, the age structure of the fish population has not been accounted for in the current model formulation. The fish population can indeed be split into age-specific classes (e.g. juveniles and adults), each with its own epidemiological compartments with different mortality, recovery and infection rates. To better elucidate this aspect, experimental investigations are currently being conducted to assess possible age-structure effects on *T. bryosalmonae* spore load in freshwater systems (Strepparava, personal communication). Our epidemiological model also relies on the simplifying hypothesis that all statoblasts hatch at the beginning of the following season. Hence, another aspect needing further investigation is the possibility of the existence of a statoblast bank (as suggested by Freeland et al. [61]). A statoblast bank could form on the bed of the water body if statoblasts were able to survive and maintain their infection status for more than one season. Dormant infected statoblasts could thus trigger new PKD outbreaks even if the disease were no longer present in fish and bryozoans.

Although the described transmission processes imply proximity between spores and hosts, several mechanisms allow long-distance spreading of PKD along river networks and lakes, among which bryozoan fragmentation, buoyancy of surfaces with attached colonies, fish migration, and hydrodynamic dispersal of *T. bryosalmonae* spores and bryozoan statoblasts may play a remarkable role [19]. To understand the role played by spatial processes in PKD transmission, the local model presented herein should be extended by considering the hydrological connectivity among different river reaches. In particular, modelling efforts at the river network scales (corroborated by field analyses) should try to understand whether PKD transmission is a spatially diffuse process or rather concentrated in transmission hot-spots. Such hot-spots could be represented by favorable habitats for both salmonids and bryozoans. The proximity of the hosts in these habitats could promote high transmission rates that may in turn be able to sustain the infection at the network scale through dispersal of fish and hydrodynamic transport of parasite spores. The possible identification of such hot-spots could offer alternative strategies to control the disease in the wild, like the confinement or the removal of one of the hosts in the isolated hot-spots.

## Conclusions

We have developed a discrete-continuous hybrid epidemiological model that incorporates the current knowledge about *T. bryosalmonae* life-cycle and PKD transmission in wild salmonid populations. Our analysis showed that, for realistic parameter ranges, parasite invasion and long-term establishment are very likely, leading to a high risk of PKD outbreaks in susceptible environments. When the disease is endemic, the impact of PKD outbreaks is shown to be mostly controlled by the rates at which the disease develops in the fish population. These parameters can be estimated via experimental and field studies. Further studies are needed to gain additional insights on key disease dynamics, in particular concerning possible recovery in both hosts.

## Additional files

Additional file 1: Linear stability analysis of the within-season system. (PDF 1644 kb) Additional file 2: MATLAB script performing simulations of the epidemiological model. It consists of 4 files. (ZIP 24 kb)

## Abbreviations

DFE: Disease free equilibrium
DFT: Disease free trajectory
PKD: Proliferative kidney disease

## References

[1] Altizer S, Ostfeld RS, Johnson PTJ, Kutz S, Harvell CD (2013). Climate change and infectious diseases: from evidence to a predictive framework. Science.

[2] Harvell CD, Mitchell CE, Ward JR, Altizer S, Dobson AP, Ostfeld RS, Samuel MD (2002). Climate warming and disease risks for terrestrial and marine biota. Science.

[3] Raffel TR, Romansic JM, Halstead NT, McMahon TA, Venesky MD, Rohr JR (2013). Disease and thermal acclimation in a more variable and unpredictable climate. Nat Clim Chang.

[4] Hedrick RP, MacConnell E, de Kinkelin P (1993). Proliferative kidney disease of salmonid fish. Annu Rev Fish Dis.

[5] Clifton-Hadley RS, Richards RH, Bucke D (1986). Proliferative kidney disease (PKD) in rainbow trout *Salmo gairdneri*: further observations on the effects of water temperature. Aquaculture.

[6] Feist SW, Longshaw M, Woo PTK (2006). Phylum Myxozoa. Fish Diseases and Disorders.

[7] Burkhardt-Holm P, Giger W, Güttinger H, Ochsenbein U, Peter A, Scheurer K (2005). Where have all the fish gone?. Environ Sci Technol.

[8] Wahli T, Bernet D, Steiner PA, Schmidt-Posthaus H (2007). Geographic distribution of *Tetracapsuloides bryosalmonae* infected fish in Swiss rivers: an update. Aquat Sci.

[9] Wahli T, Knuesel R, Bernet D, Segner H, Pugovkin D, Burkhardt-Holm P (2002). Proliferative kidney disease in Switzerland: current state of knowledge. J Fish Dis.

[10] Anderson CL, Canning EU, Okamura B (1999). Molecular data implicate bryozoans as hosts for PKX (Phylum Myxozoa) and identify a clade of bryozoan parasites within the Myxozoa. Parasitology.

[11] Longshaw M, Feist SW, Canning EU, Okamura B (1999). First identification of PKX in bryozoans from the United Kingdom - molecular evidence. Bull Eur Assoc Fish Pathol.

[12] Okamura B, Anderson CL, Longshaw M, Feist SW, Canning EU (2001). Patterns of occurrence and 18 s rDNA sequence variation of PKX (*Tetracapsula bryosalmonae*), the causative agent of salmonid proliferative kidney disease. J Parasitol.

[13] Feist SW, Bucke D (1993). Proliferative kidney disease in wild salmonids. Fish Res.

[14] Bettge K, Segner H, Burki R, Schmidt-Posthaus H, Wahli T (2009). Proliferative kidney disease (PKD) of rainbow trout: temperature- and time-related changes of *Tetracapsuloides bryosalmonae* DNA in the kidney. Parasitol.

[15] Bettge K, Wahli T, Segner H, Schmidt-Posthaus H (2009). Proliferative kidney disease in rainbow trout: time- and temperature-related renal pathology and parasite distribution. Dis Aquat Organ.

[16] Ferguson HW, Ball HJ (1979). Epidemiological aspects of proliferative kidney disease amongst rainbow trout *Salmo gairdneri* Richardson in Northern Ireland. J Fish Dis.

[17] Tops S, Hartikainen H, Okamura B (2009). The effects of infection by *Tetracapsuloides bryosalmonae* (Myxozoa) and temperature on *Fredericella sultana* (Bryozoa). Int J Parasitol.

[18] Tops S, Lockwood W, Okamura B (2006). Temperature-driven proliferation of *Tetracapsuloides bryosalmonae* in bryozoan hosts portends salmonid declines. Dis Aquat Organ.

[19] Okamura B, Hartikainen H, Schmidt-Posthaus H, Wahli T (2011). Life cycle complexity, environmental change and the emerging status of salmonid proliferative kidney disease. Freshwat Biol.

[20] Anderson RM, May RM (1991). Infectious diseases of humans: dynamics and control.

[21] Keeling MJ, Rohani P (2007). Modeling infectious diseases in humans and animals.

[22] Revie CW, Robbins C, Gettinby G, Kelly L, Treasurer JW (2005). A mathematical model of the growth of sea lice, *Lepeophtheirus salmonis*, populations on farmed Atlantic salmon, *Salmo salar* L., in Scotland and its use in the assessment of treatment strategies. J Fish Dis.

[23] Taylor NG, Norman RA, Way K, Peeler EJ (2011). Modelling the koi herpesvirus (KHV) epidemic highlights the importance of active surveillance within a national control policy. J Appl Ecol.

[24] Masson I, Lotz JM, Blaylock RB (2013). Population model for *Amyloodinium ocellatum* infecting the spotted seatrout *Cynoscion nebulosus* and the red snapper *Lutjanus campechanus*. Dis Aquat Organ.

[25] Boerlage AS, Graat EAM, Verreth JA, de Jong MCM (2013). Effect of control strategies on the persistence of fish-borne zoonotic trematodes: a modelling approach. Aquaculture.

[26] Ray RA. Modeling abiotic influences on disease dynamics for the complex life cycle of the myxozoan parasite *Ceratomyxa shasta*. Ph.D. thesis, Oregon State University. 2013.

[27] Turner KG, Smith MJ, Ridenhour BJ (2014). Whirling disease dynamics: an analysis of intervention strategies. Prev Vet Med.

[28] Borsuk ME, Reichert P, Peter A, Schager E, Burkhardt-Holm P (2006). Assessing the decline of brown trout (*Salmo trutta*) in Swiss rivers using a Bayesian probability network. Ecol Model.

[29] Hartikainen H, Okamura B, Okamura B, Gruhl A, Bartholomew JL (2015). Ecology and evolution of malacosporean-bryozoan interactions. Myxozoan Evolution, Ecology and Development.

[30] Canning EU, Tops S, Curry A, Wood TS, Okamura B (2002). Ecology, development and pathogenicity of *Buddenbrockia plumatellae* Schröder, 1910 (Myxozoa, Malacosporea) (syn. *Tetracapsula bryozoides*) and establishment of *Tetracapsuloides* n. gen. for *Tetracapsula bryosalmonae*. J Eukaryot Microbiol.

[31] Morris DJ, Adams A (2006). Proliferative, presaccular stages of *Tetracapsuloides bryosalmonae* (Myxozoa: Malacosporea) within the invertebrate host *Fredericella sultana* (Bryozoa: Phylactolaemata). J Parasitol.

[32] Canning EU, Curry A, Feist SW, Longshaw M, Okamura B (2000). A new class and order of myxozoans to accommodate parasites of bryozoans with ultrastructural observations on *Tetracapsula bryosalmonae* (PKX organism). J Eukaryot Microbiol.

[33] Canning EU, Curry A, Feist SW, Longshaw M, Okamura B (1999). *Tetracapsula bryosalmonae* n.sp. for PKX organism, the cause of PKD in salmonid fish. Bull Eur Assoc Fish Pathol.

[34] Tops S. Ecology, life history and diversity of malacosporeans. Ph.D. thesis, University of Reading. 2004.

[35] Hartikainen H, Johnes P, Moncrieff C, Okamura B (2009). Bryozoan populations reflect nutrient enrichment and productivity gradients in rivers. Freshwat Biol.

[36] Hartikainen H, Okamura B (2012). Castrating parasites and colonial hosts. Parasitology.

[37] Abd-Elfattah A, Fontes I, Kumar G, Soliman H, Hartikainen H, Okamura B (2014). Vertical transmission of *Tetracapsuloides bryosalmonae* (Myxozoa), the causative agent of salmonid proliferative kidney disease. Parasitology.

[38] Feist SW, Longshaw M, Canning EU, Okamura B (2001). Induction of proliferative kidney disease (PKD) in rainbow trout *Oncorhynchus mykiss* via the bryozoan *Fredericella sultana* infected with *Tetracapsula bryosalmonae*. Dis Aquat Organ.

[39] Longshaw M, Le Deuff RM, Harris AF, Feist SW (2002). Development of proliferative kidney disease in rainbow trout, *Oncorhynchus mykiss* (Walbaum), following short-term exposure to *Tetracapsula bryosalmonae* infected bryozoans. J Fish Dis.

[40] Kent ML, Hedrick RP (1985). PKX, the causative agent of proliferative kidney disease (PKD) in Pacific salmonid fishes and its affinities with the Myxozoa. J Protozool.

[41] Morris DJ, Adams A (2008). Sporogony of *Tetracapsuloides bryosalmonae* in the brown trout *Salmo trutta* and the role of the tertiary cell during the vertebrate phase of myxozoan life cycles. Parasitology.

[42] Hedrick RP, Baxa DV, De Kinkelin P, Okamura B (2004). Malacosporean-like spores in urine of rainbow trout react with antibody and DNA probes to *Tetracapsuloides bryosalmonae*. Parasitol Res.

[43] Abd-Elfattah A, Kumar G, Soliman H, El-Matbouli M (2014). Persistence of *Tetracapsuloides bryosalmonae* (Myxozoa) in chronically infected brown trout *Salmo trutta*. Dis Aquat Organ.

[44] Grabner DS, El-Matbouli M (2008). Transmission of *Tetracapsuloides bryosalmonae* (Myxozoa: Malacosporea) to *Fredericella sultana* (Bryozoa: Phylactolaemata) by various fish species. Dis Aquat Organ.

[45] Morris DJ, Adams A (2006). Transmission of *Tetracapsuloides bryosalmonae* (Myxozoa: Malacosporea), the causative organism of salmonid proliferative kidney disease, to the freshwater bryozoan *Fredericella sultana*. Parasitology.

[46] Ricker WE (1954). Stock and recruitment. J Fish Res Board Can.

[47] Agarwal R, Bohner M, O’Regan D, Peterson A (2002). Dynamic equations on time scales: a survey. J Comput Appl Math.

[48] El-Matbouli M, Hoffman RW, Pike AW, Lewis JW (1994). Proliferative kidney disease (PKD) as an important myxosporean infection in salmonid fish. Parasitic Diseases of Fish.

[49] Foott JS, Hedrick RP (1987). Seasonal occurrence of the infectious stage of proliferative kidney disease (PKD) and resistance of rainbow trout, *Salmo gairdneri* Richardson, to reinfection. J Fish Biol.

[50] Bacaër N, Ouifki R (2007). Growth rate and basic reproduction number for population models with a simple periodic factor. Math Biosci.

[51] Mari L, Casagrandi R, Bertuzzo E, Rinaldo A, Gatto M (2014). Floquet theory for seasonal environmental forcing of spatially explicit waterborne epidemics. Theor Ecol.

[52] Wang W, Zhao X (2008). Threshold dynamics for compartmental epidemic models in periodic environments. J Dyn Differ Equ.

[53] Guckenheimer J, Holmes P (1983). Nonlinear Oscillations, Dynamical Systems and Bifurcation of Vector Fields, Applied Mathematical Sciences, volume 42.

[54] Morris DJ, Adams A, Feist SW, McGeorge J, Richards RH (2000). Immunohistochemical and PCR studies of wild fish for *Tetracapsula bryosalmonae* (PKX), the causative organism of proliferative kidney disease. J Fish Dis.

[55] Schmidt-Posthaus H, Bettge K, Forster U, Segner H, Wahli T (2012). Kidney pathology and parasite intensity in rainbow trout Oncorhynchus mykiss surviving proliferative kidney disease: time course and influence of temperature. Dis Aquat Organ.

[56] Kumar G, Abd-Elfattah A, Saleh M, El-Matbouli M (2013). Fate of *Tetracapsuloides bryosalmonae* (Myxozoa) after infection of brown trout *Salmo trutta* and rainbow trout *Oncorhynchus mykiss*. Dis Aquat Organ.

[57] Grabner DS, El-Matbouli M (2009). Comparison of the susceptibility of brown trout (*Salmo trutta*) and four rainbow trout (*Oncorhynchus mykiss*) strains to the myxozoan *Tetracapsuloides bryosalmonae*, the causative agent of proliferative kidney disease (PKD). Vet Parasitol.

[58] Elith J, Leathwick JR (2009). Species distribution models: ecological explanation and prediction across space and time. Annu Rev Ecol Evol Syst.

[59] Økland KA, Økland J (2001). Freshwater bryozoans (bryozoa) of Norway ii: distribution and ecology of two species of *fredericella*. Hydrobiologia.

[60] Schager E, Peter A, Burkhardt-Holm P (2007). Status of young-of-the-year brown trout (*Salmo trutta fario*) in Swiss streams: factors influencing YOY trout recruitment. Aquat Sci.

[61] Freeland JR, Rimmer VK, Okamura B (2001). Genetic changes within freshwater bryozoan populations suggest temporal gene flow from statoblast banks. Limnol Oceanogr.

[62] De Kinkelin P, Gay M, Forman S (2002). The persistence of infectivity of *Tetracapsula bryosalmonae*-infected water for rainbow trout, *Oncorhynchus mykiss* (Walbaum). J Fish Dis.

[63] Ayllón D, Almodóvar A, Nicola GG, Parra I, Elvira B (2012). Modelling carrying capacity dynamics for the conservation and management of territorial salmonids. Fish Res.

