# Supplementary material for: An epidemiological model for proliferative kidney disease in salmonid populations
Source: Parasit Vectors. 2016 Sep 5;9(1):487. doi: 10.1186/s13071-016-1759-z (PMC5011885; doi:10.1186/s13071-016-1759-z)
Supplement: Additional file 1: — Linear stability analysis of the within-season system. (PDF 1644 kb) [file 13071_2016_1759_MOESM1_ESM.pdf]

Linear stability analysis of the within-season system

We consider a within-season system derived from Eqs. (1) and (3). In such a system, we first neglect the equations expressing the time evolution of statoblasts, because statoblasts only affect other state variables in the transition between two seasons. Moreover, in order to define a disease-free equilibrium, we neglect the equation related to the time evolution of susceptible bryozoan biomass and susceptible fish abundance and treat the terms B_S and F_S appearing in other equations as parameters. All parameters are assumed as constant in time. Hence, this system reads:

$$\frac{dB_C}{d\tau} = r\left[1-\rho(B_S+B_C+B_O)\right]B_C-(d_{CO}+\psi)B_C+d_{OC}B_O+B_S Z_F^*; \tag{A1a}$$

$$\frac{dB_O}{d\tau} = r_O\left[1-\rho(B_S+B_C+B_O)\right]B_O+d_{CO}B_C-(d_{OC}+\psi)B_O; \tag{A1b}$$

$$\frac{dF_E}{d\tau} = -(\mu_F+h)F_E+F_S Z_B^*; \tag{A1c}$$

$$\frac{dF_I}{d\tau} = (1-\epsilon)hF_E-(\mu_F+a+\gamma)F_I; \tag{A1d}$$

$$\frac{dF_c}{d\tau} = \epsilon hF_E+\gamma F_I-(\mu_F+\zeta)F_C; \tag{A1e}$$

$$\frac{dZ_B}{d\tau} = \pi_B^* B_O-\mu_Z Z_B^*; \tag{A1f}$$

$$\frac{dZ_F}{d\tau} = \pi_F^*(F_I+\kappa F_C)-\mu_Z Z_F^*. \tag{A1g}$$

The disease-free equilibrium (DFE) of the system (A1) is $\mathbf{x_{DF}} = \{B_C; B_O; F_E; F_I; F_C; Z_B^*; Z_F^*\} = \mathbf{0}$. We now linearise (A1) around $\mathbf{x_{DF}}$ and calculate its Jacobian:

$$
\mathbf{J_{DF}} = \begin{bmatrix}
J_{DF,11} & d_{OC} & 0 & 0 & 0 & 0 & B_S \\
d_{CO} & J_{DF,22} & 0 & 0 & 0 & 0 & 0 \\
0 & 0 & -h-\mu_F & 0 & 0 & F_S & 0 \\
0 & 0 & (1-\epsilon)h & -a-\gamma-\mu_F & 0 & 0 & 0 \\
0 & 0 & \epsilon h & \gamma & -\zeta-\mu_F & 0 & 0 \\
0 & \pi_B^* & 0 & 0 & 0 & -\mu_Z & 0 \\
0 & 0 & 0 & \pi_F^* & \kappa\pi_F^* & 0 & -\mu_Z
\end{bmatrix}.
$$

where $J_{DF,11} = r(1-\rho B_S) - \psi - d_{CO}$ and $J_{DF,22} = r_O(1-\rho B_S) - \psi - d_{OC}$.

According to a classical result of dynamical system theory [1], the spectral abscissa $\alpha_s(\mathbf{J_{DF}})$ of the Jacobian of (A1) expresses the speed at which a perturbation of the equilibrium state will propagate; if $\alpha_s(\mathbf{J_{DF}}) < 0$, the DFE is stable.

The determinant of $\mathbf{J_{DF}}$ reads:

$$
\det(\mathbf{J_{DF}}) = J_1 - J_2\mathcal{T}
$$

where

$$
J_1 = B_S F_S d_{CO} h \pi_B^* \pi_F^* \left[\epsilon\kappa a + \kappa\gamma + (1-\epsilon)\zeta + (1+\epsilon\kappa-\epsilon)\mu_F\right];
$$

$$
J_2 = (h+\mu_F)(a+\gamma+\mu_F)(\zeta+\mu_F)\mu_Z^2;
$$

$$
\mathcal{T} = \psi^2 + \left[d_{CO} + d_{OC} - (r+r_O)(1-\rho B_S)\right]\psi - (1-\rho B_S)\left[r_O d_{CO} + r d_{OC} + r r_O(1-\rho B_S)\right].
$$

Note that J_1 and J_2 are non-negative by construction. Given that the order of $\mathbf{J_{DF}}$ is odd, a switch in the sign of $\alpha_s(\mathbf{J_{DF}})$ from negative to positive corresponds to a switch in the sign of $\det(\mathbf{J_{DF}})$ from negative to positive. Also, $\det(\mathbf{J_{DF}}) > 0$ is a sufficient condition for the instability of the $\mathbf{x_{DF}}$. This condition is always true when $\mathcal{T} < 0$: in this case, the parasite can spread within the bryozoan population even in the absence of the fish population (i.e. when $F_S = 0$ and thus $J_1 = 0$).

Indeed, it is straightforward to show that $\mathcal{T}$ is the determinant of the Jacobian of a system that considers only bryozoans (i.e. Eqs. (A1a) with $Z_F^* = 0$ and (A1b)), with $\mathcal{T} < 0$ representing the instability criterion for the DFE of such a system. If instead $\det(\mathbf{J_{DF}}) > 0$ with $\mathcal{T} > 0$, $\mathbf{x_{DF}}$ is still unstable, but in this case the parasite needs to cycle between the two hosts to invade the system.

A condition of incipient instability can be derived when $\det(\mathbf{J_{DF}}) = 0$, which leads to an expression for the reproduction number $\mathcal{R}$:

$$\mathcal{R} = \frac{J_1}{J_2 \mathcal{T}}. \tag{A2}$$

The DFE is unstable when $\mathcal{R}$ is larger than unity. Note that the expression (A2) is only valid if $\mathcal{T} > 0$; failing that, the DFE is always unstable and $\mathcal{R}$ can not be defined.

Figure A1 identifies regions of stability and instability for the disease-free equilibrium in the parameter space.

References

[1] Luenberger, David G. (1979). Introduction to dynamic systems: theory, models, and applications. Wiley. ISBN 978-0-471-02594-8.

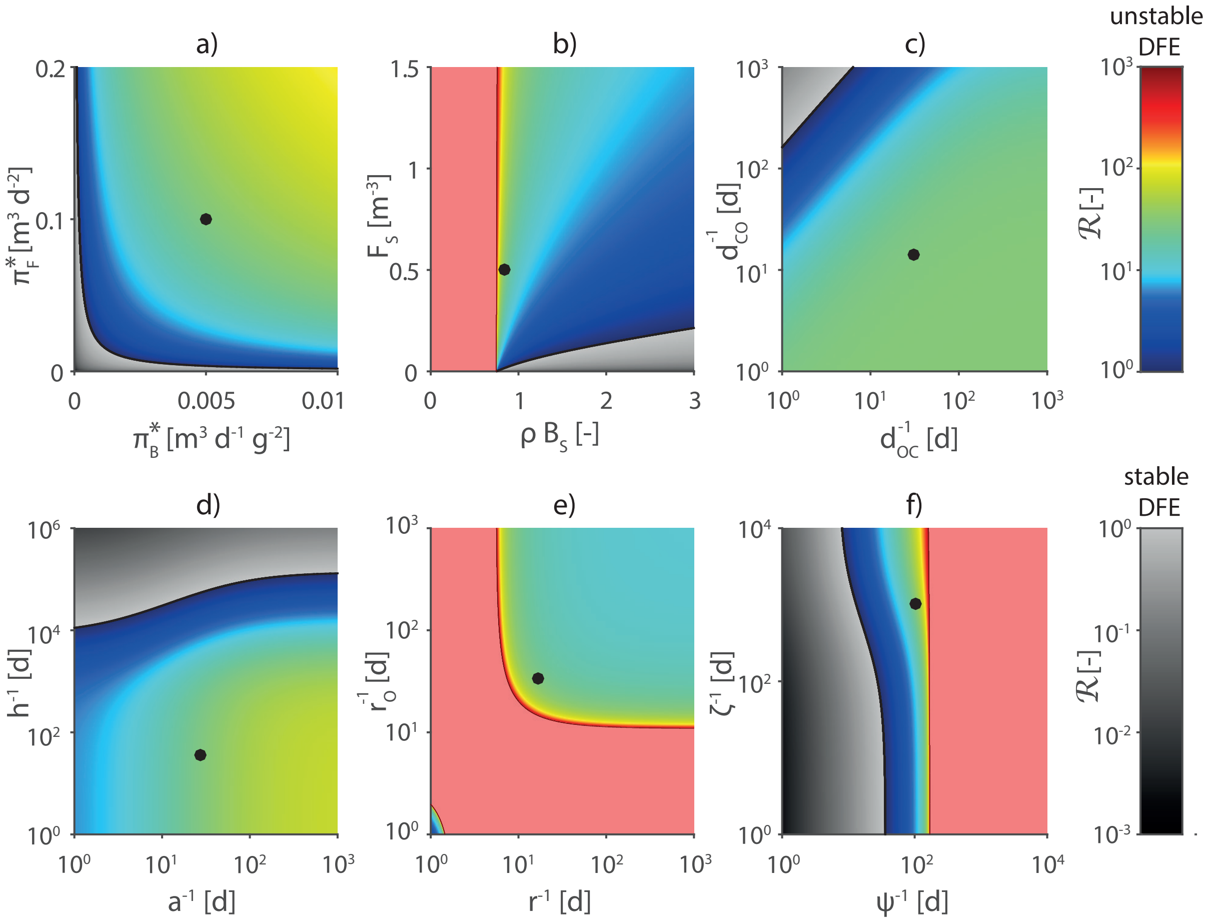

Figure A1: Values of the reproductive number $\mathcal{R}$ of system (A1) as a function of the model parameters. Two parameters are varied at a time, the others are assumed as in Table 3. Regions where $\mathcal{T} < 0$ (DFE unstable) are coloured in light red. Black dots refer to the reference parameter set. All rates are expressed as mean times (i.e., by their inverse). With regards to temperature-dependent parameters, their value at 15 °C is displayed.
